# Development and validation of a novel biomarker panel for Crohn’s disease and rheumatoid arthritis diagnosis and treatment

**DOI:** 10.18632/aging.205644

**Published:** 2024-03-10

**Authors:** Hao Zhang, Wenhao Qiao, Ran Liu, Zuoxiu Shi, Jie Sun, Shuxiao Dong

**Affiliations:** 1 Department of Gastroenterology Surgery, Shandong Provincial Third Hospital, Shandong University, Jinan, Shandong 250013, China

**Keywords:** Crohn’s disease, rheumatoid arthritis, biomarker panel, immune infiltration, drug prediction

## Abstract

Background: Crohn’s disease (CD) and rheumatoid arthritis (RA) are immune-mediated inflammatory diseases. However, the molecular mechanisms linking these two diseases remain unclear.

Methods: To identify shared core genes between CD and RA, we employed differential gene analysis and the least absolute shrinkage and selection operator (LASSO) algorithm. Functional annotation of these core biomarkers was performed using consensus clustering and gene set enrichment analysis. We also constructed a protein-protein network and a miRNA-mRNA network using multiple databases, and potential therapeutic agents targeting the core biomarkers were predicted. Finally, we confirmed the expression of the genes in the biomarker panel in both CD and RA using quantitative PCR.

Results: A total of five shared core genes, namely C-X-C motif chemokine ligand 10 (CXCL10), C-X-C motif chemokine ligand 9 (CXCL9), aquaporin 9 (AQP9), secreted phosphoprotein 1 (SPP1), and metallothionein 1M (MT1M), were identified as core biomarkers. These biomarkers activate classical pro-inflammatory and immune signaling pathways, influencing immune cell aggregation. Additionally, testosterone was identified as a potential therapeutic agent targeting the biomarkers identified in this study. The expression of genes in the biomarker panel in CD and RA was confirmed through quantitative PCR.

Conclusion: Our study revealed some core genes shared between CD and RA and established a novel biomarker panel with potential implications for the diagnosis and treatment of these diseases.

## INTRODUCTION

Crohn’s disease (CD) is an immune-mediated, chronic inflammatory disease of the gastrointestinal tract that may lead to progressive gut dysfunction and long-term disability [1, 2]. Although the etiology of CD remains unclear, recent therapeutic approaches have shifted from mere symptom management to prioritizing the attainment of clinical and deep remission [3]. Indeed, the exploration of complex molecular mechanisms associated with CD and the identification of reliable biomarkers for diagnosis and treatment are crucial endeavors in the field of clinical management.

Rheumatoid arthritis (RA) is an immune-mediated inflammatory disease often treated with medications frequently used for CD, such as infliximab and adalimumab [4]. CD and RA appear to be interconnected, as patients with these conditions often exhibit similar pathological alterations mediated by shared molecular mechanisms [5]. Hence, we contend that investigating the genes common to both diseases can potentially yield valuable insights for enhancing disease diagnosis and advancing the development of therapeutic drugs.

In this study, we analyzed transcriptomic data of CD and RA to identify shared core biomarkers. Subsequently, we investigated the mechanism by which these biomarkers influence molecular signaling pathways and their effect on immune cell infiltration. We validated our findings in an independent external cohort of patients, confirming the biomarkers’ specificity to CD and RA. Finally, we constructed microRNA (miRNA)−mRNA co-expression networks and protein–protein interaction networks for these biomarkers, leading to the identification of potential therapeutic agents for the treatment of both CD and RA.

In summary, this study offers novel insights into the diagnosis and treatment of CD and RA by elucidating the shared molecular mechanisms that underlie their pathogenesis.

## MATERIALS AND METHODS

### Data acquisition and preparation

The datasets utilized in this study were obtained from the Gene Expression Omnibus (GEO) database. We employed the keywords “Crohn’s disease” and “rheumatoid arthritis” as search terms, excluding all datasets involving treatment measures. To further mitigate the effect of a single sample source, we selected CD datasets (GSE75214 and GSE102133, platform = “GPL570”) and RA datasets (GSE55457 and GSE55235, platform = “GPL96”) from distinct institutions while ensuring they were based on the same microarray platform as the discovery cohort. Subsequently, the datasets in the discovery cohort were merged separately using the R packages “dplyr” and “sva” to eliminate batch effects. Unsupervised principal component analysis (PCA) was then performed on the discovery cohort using the R package “ggbiplot.”

The GSE16879, GSE20881, and GSE179285 datasets served as validation cohorts for CD, while the GSE77298 dataset served as the validation cohort for RA. Additionally, the GSE48958 dataset for ulcerative colitis (UC) and the GSE82107 dataset for arthritis were used as supplementary control cohorts for other diseases. In addition, in cases where the dataset’s maximum value exceeded 100, the data were log-transformed using the following formula: log_2_(x+1). Detailed information about the datasets used in this study is provided in Table 1.

**Table 1:** Information from the microarray dataset included in this study.

**GEO accession**	**Platform**	**Samples**	**Source tissue**	**Attribute**
GSE75214	GPL6244	75 CD and 22 Normal	Mucosal	Discovery cohort
GSE102133	GPL6244	65 CD and 12 Normal	Mucosal	Discovery cohort
GSE55235	GPL96	10 RA and 10 Normal	Synovial	Discovery cohort
GSE55457	GPL96	13 RA and 10 Normal	Synovial	Discovery cohort
GSE48958	GPL6244	13 UC and 8 Normal	Mucosal	Validation cohort
GSE82107	GPL570	13 OA and 7 Normal	Synovial	Validation cohort
GSE16879	GPL570	73 CD and 12 Normal	Mucosal	Validation cohort
GSE20881	GPL1708	99 CD and 73 Normal	Mucosal	Validation cohort
GSE179285	GPL6480	168 CD and 31 Normal	Mucosal	Validation cohort
GSE77298	GPL570	16 RA and 7 Normal	Synovial	Validation cohort

Abbreviations: GEO: Gene Expression Omnibus; CD: Crohn’s disease; RA: Rheumatoid arthritis; UC: ulcerative colitis; OA: osteoarthritis.

### Identification of differentially expressed genes (DEGs)

Differential gene analysis was performed using the R package “limma.” The criteria for identifying DEGs were defined as a |log_2_ fold change| > 1 with a *P*-value <0.05. Volcano plots and heatmaps depicting the DEGs were generated using the “ggplot2” and “ggpheatmap” functions. The DEGs that were observed in both the CD and RA datasets were identified and represented visually using the R package “ggVennDiagram.”

### Identification of core DEGs

The shared DEGs in both the CD and RA discovery cohorts were identified using the least absolute shrinkage and selection operator (LASSO) algorithm with 10-fold cross-validation. The most optimal gene combinations were identified based on the minimum lambda values. This analysis was conducted using the R package “glmnt,” and the results were visually represented using the R package “ggplot2.” The most optimal biomarker combinations from the CD and RA discovery cohorts were intersected to identify common core biomarkers for CD and RA, and the results were visually presented using the R package “ggVennDiagram.”

### Consensus clustering

Using the identified biomarkers, we performed a consensus clustering analysis based on resampling in the discovery cohort. This analysis was executed using the “ConsensusClusterPlus” package in R [6]. Consensus score matrices and cumulative distribution function (CDF) curves were employed to determine the optimal number of clusters in the CD and RA discovery cohorts. Finally, we considered the optimal number of clusters for both the CD and RA discovery cohorts as k = 2 and employed this to divide the discovery cohort into two distinct subtypes, referred to as “cluster 1” and “cluster 2.”

### Gene function annotation enrichment analysis

Gene ontology (GO) and Kyoto Encyclopedia of Genes and Genomes (KEGG) enrichment analyses were performed on the DEGs among the various subtypes. Subsequently, the biological processes and signaling pathways associated with the biomarker panel were identified. To assess the associations between the biomarker panel and these signaling pathways, we employed gene set enrichment analysis (GSEA). This assessment was conducted using the R package “clusterProfiler” [7], and the results were visualized with the R packages “ggplot2” and “GseaVis.”

### Immune infiltration analysis

Immune infiltration analysis of the CD and RA discovery cohorts was performed employing single-sample GSEA (ssGSEA) [8], ESTIMATE, and CIBERSORT [9]. In this study, the ssGSEA immune score was defined as the sum of all immune cell scores within the same sample species. This procedure was executed using the R packages “GSVA” and “estimate,” along with the “CIBERSORT” function.

### Construction of a protein–protein interaction network

We obtained a list of protein–protein interactions among the five genes in the biomarker panel from the STRING database (https://cn.string-db.org/) [10], which was employed to construct a protein–protein interaction network using the GeneMANIA database (http://genemania.org/) [11].

### Construction of an miRNA–mRNA co-expression network

To target the five genes constituting the shared core biomarkers between CD and RA, we identified the miRNAs capable of targeting these genes employing data from three databases: TargetScan (https://www.targetscan.org/vert_80/) [12], miRWalk (http://mirwalk.umm.uni-heidelberg.de/), and miRDB (https://mirdb.org/) [13]. Subsequently, we constructed and visualized the miRNA-RNA co-expression network using Cytoscape 3.8.2 software [14].

### Identification of potential therapeutic compounds and molecular docking

We identified compounds targeting the five genes in the biomarker panel using the Drug Gene Interaction (DGI) database (https://dgidb.org/) [15]. Subsequently, we constructed and visualized the action networks of the identified compounds using Cytoscape. Information regarding the structures of the compounds and their respective target proteins was obtained from PubChem (https://pubchem.ncbi.nlm.nih.gov/) and the PDB database (https://www.rcsb.org/), respectively. Molecular docking was performed using Autodock Vina [16] with an exhaustiveness value set at 10. The Grix box centers for testosterone (PubChem CID: 6013) and C-X-C motif chemokine ligand 10 (CXCL10) (PDB ID: 1LV9) were positioned at *x* = 4.028, *y* = −0.351, and *z* = −4.536, with a Grix box size of *x* = 36.0, *y* = 23.25, and *z* = 21.75. Additionally, the Grix box centers for testosterone in conjunction with aquaporin 9 (AQP9) (PDB ID: 6QZJ) were set at *x* = −6.926, *y* = 11.231, and *z* = −119.111, with a Grix box size of *x* = 126, *y* = 126, and *z* = 126. The results of the molecular docking were visualized utilizing PyMOL 2.2.0 software.

### Quantitative PCR (q-PCR)

As previously reported [17], a q-PCR assay was employed to assess alterations in the mRNA expression of *CXCL10*, *CXCL9*, *SPP1* (secreted phosphoprotein 1), *AQP9*, and *MT1M* (metallothionein 1M). Briefly, total RNA was extracted from frozen mouse colonic mucosal tissues using TRIzol reagent (Invitrogen, Carlsbad, CA, USA), and subsequently, purified complementary DNA was synthesized through reverse transcription of the isolated RNA. The Synergistic Branding (SYBR) Green assay (Applied Biosystems, Carlsbad, CA, USA) was employed for quantifying target gene transcription, according to the manufacturer’s protocol. The relative expression of the target genes, normalized to the expression of glyceraldehyde-3-phosphate dehydrogenase, was determined using the 2^−ΔΔCT^ method. The sequences of the primers used in the assay are provided in Table 2.

**Table 2:** Sequences of primers used for q-PCR.

**Gene**	**Forward primer**	**Reverse primer**
*CXCL10*	AATCATCCCTGCGAGCCTATCC	TGTGCGTGGCTTCACTCCAGTT
*CXCL9*	CATCATCTTCCTGGAGCAGTGTGG	AGTCTTCCTTGAACGACGACGAC
*SPP1*	CAAACGCCGACCAAGGAAAA	GGCCACAGCATCTGGGTATT
*AQP9*	CCCAGCTGTGTCTTTAGCAA	AAGTCCATCATAGTAAATGCCAAA
*MT1M*	AATAGAACAAGCTGCACAAC	TGGCTCAGTATCGTATTGAA
*GAPDH*	ATGACCACAGTCCATGCCATCAC	ATGCCTGCTTCACCACCTTCTTG

### Statistical analysis

Statistical analysis was conducted using R, version 4.2.0 (the R Project for Statistical Computing). Given that the sequencing data exhibited a non-normal distribution, all analyses in this study employed nonparametric tests. Group comparisons were performed using the unpaired Wilcoxon test, while correlation analysis was performed using the Spearman correlation. Receiver operating characteristic (ROC) curve analysis and the computation of the area under the curve (AUC) were conducted using the “pROC” package in R. A significance level of *P* < 0.05 was considered statistically significant.

### Data availability statement

The datasets used in this study were obtained from the public database Gene Expression Omnibus (https://www.ncbi.nlm.nih.gov/geo/). The numbers of all datasets have been indicated in the article.

## RESULTS

### Identification of shared DEGs in CD and RA datasets

**Figure 1:**
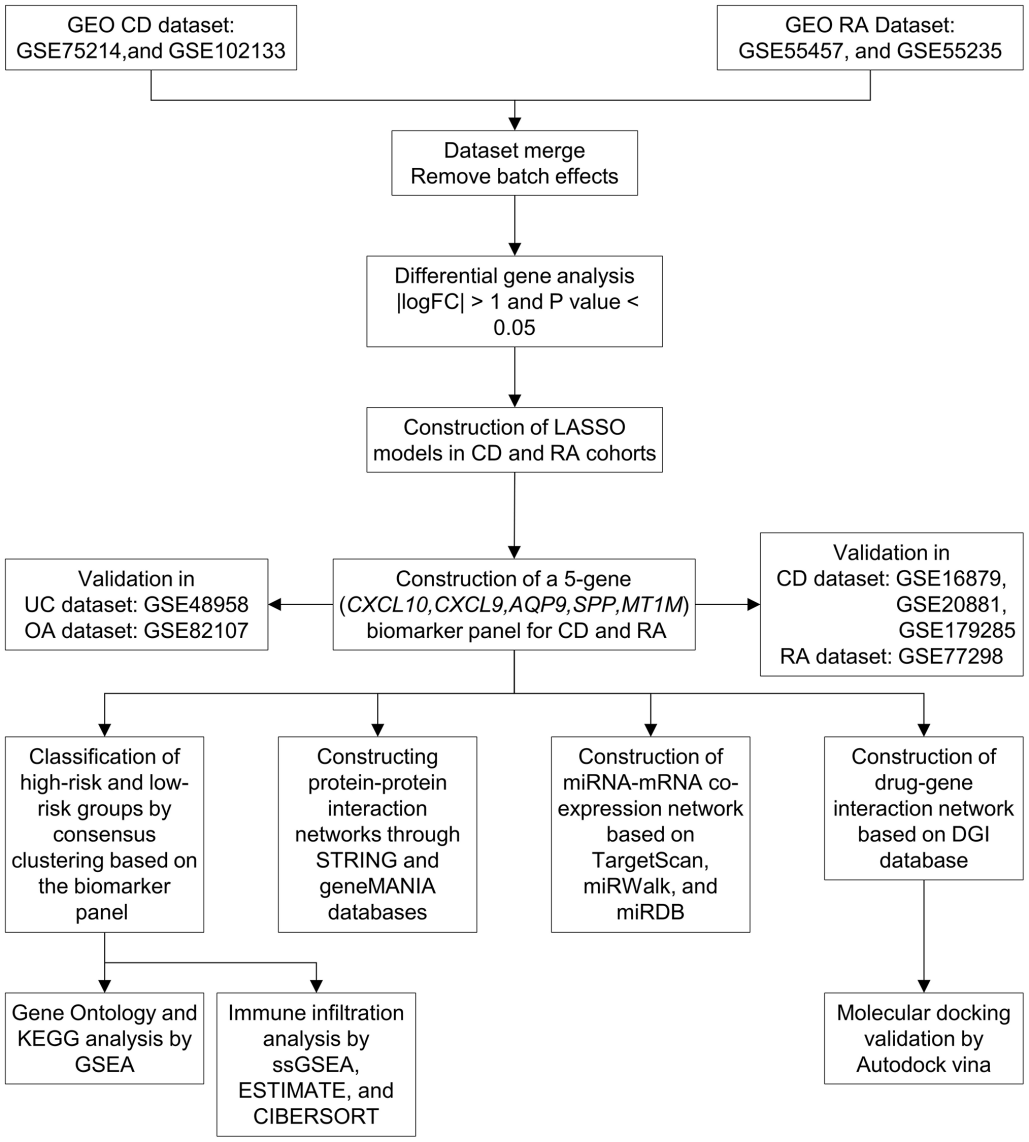
**Workflow of the study.** Abbreviations: GEO: Gene Expression Omnibus; CD: Crohn’s disease; RA: rheumatoid arthritis; LASSO: least absolute shrinkage and selection operator; UC: ulcerative colitis; OA: osteoarthritis; KEGG: Kyoto Encyclopedia of Genes and Genomes; GSEA: gene set enrichment analysis.

**Figure 2:**
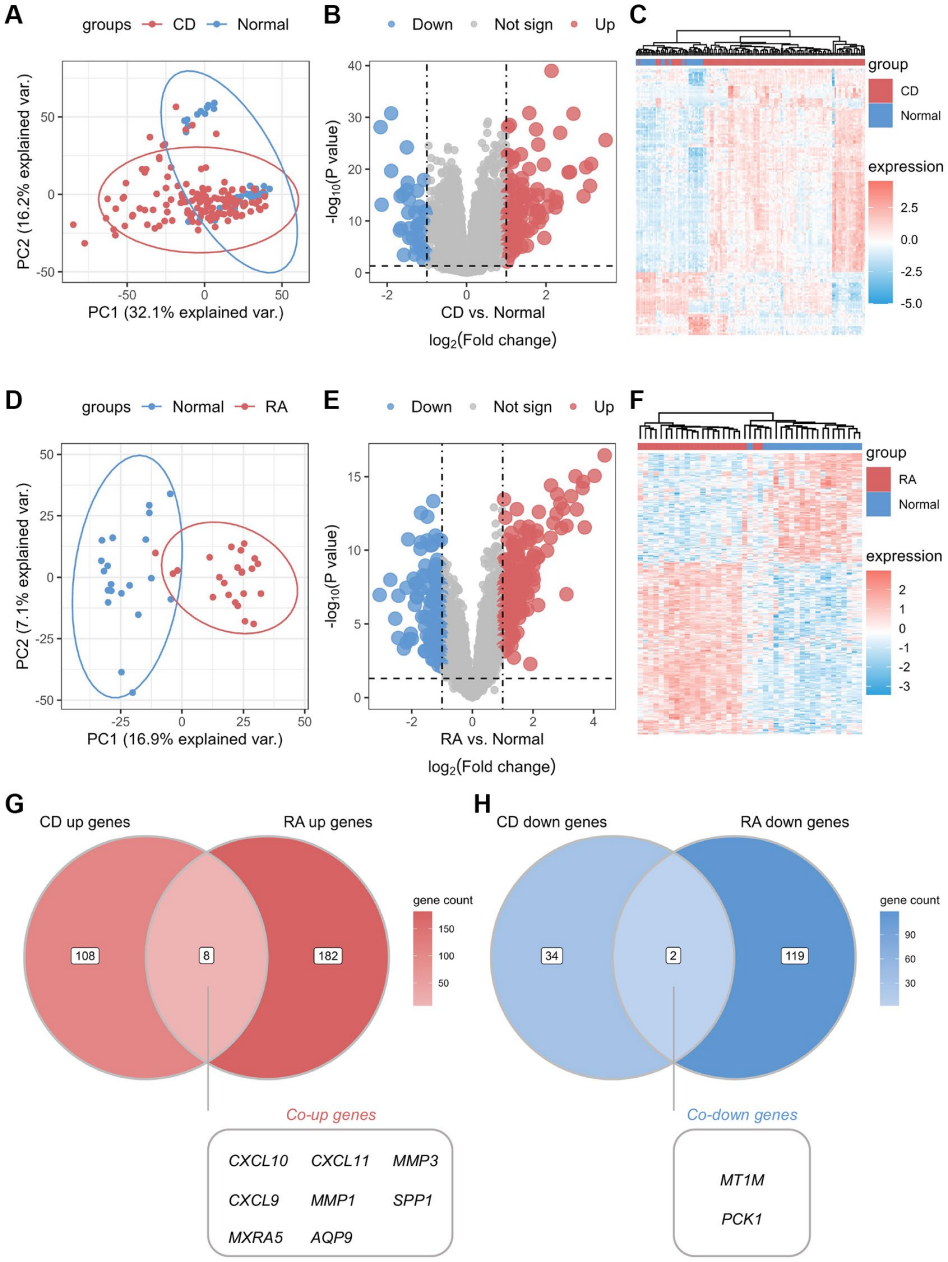
**Identification of common differential genes between CD and RA.** (**A**) Unsupervised clustering PCA plot for CD discovery cohort. (**B**) Volcano map of differential genes in the CD discovery cohort. (**C**) Unsupervised clustering heatmap of differential genes in the CD discovery cohort. (**D**) Unsupervised clustering PCA plot for the RA discovery cohort. (**E**) Volcano map of differential genes in the RA discovery cohort. (**F**) Unsupervised clustering heatmap of differential genes in the CD discovery cohort. (**G**) Venn diagram of co-upregulated differential genes in the CD and RA discovery cohorts. (**H**) Venn diagram of co-downregulated differential genes in the CD and RA discovery cohorts. Abbreviations: PCA: principal component analysis; CD: Crohn’s disease; RA: rheumatoid arthritis.

The study design flowchart is shown in Figure 1. Unsupervised clustering PCA was performed on the CD cohort, comprising the GSE75214 and GSE102133 datasets. The results revealed differences in gene expression profiles between the CD and normal groups (Figure 2A). Subsequently, DEG analysis yielded a volcano plot illustrating genes that exhibited differential expression between the CD and normal groups (Figure 2B), with an accompanying unsupervised clustering heatmap of the DEGs displayed in Figure 2C. The results showed that most DEGs were upregulated in the CD group. The analysis of the RA cohort, encompassing the GSE55457 and GSE55235 datasets, revealed significant differences in gene expression between the RA and normal groups. Moreover, these DEGs could be used to distinguish between RA and normal samples (Figure 2D–2F). When comparing the DEGs between the CD and RA cohorts, the DEGs *CXCL10*, *CXCL11*, *MMP3* (matrix metallopeptidase 3), *CXCL9*, *MMP1*, *SPP1*, *MXRA5* (matrix remodeling associated 5), and *AQP9* were found to be upregulated (Figure 2G), while the DEGs *MT1M* and *PCK1* (phosphoenolpyruvate carboxykinase 1) were observed to be downregulated in both cohorts (Figure 2H). Given the similar expression patterns of these genes in both the CD and RA cohorts, we propose that these 10 DEGs may be involved in a shared disease mechanism linking CD and RA.

### Construction of a biomarker panel for the diagnosis of CD and RA

**Figure 3:**
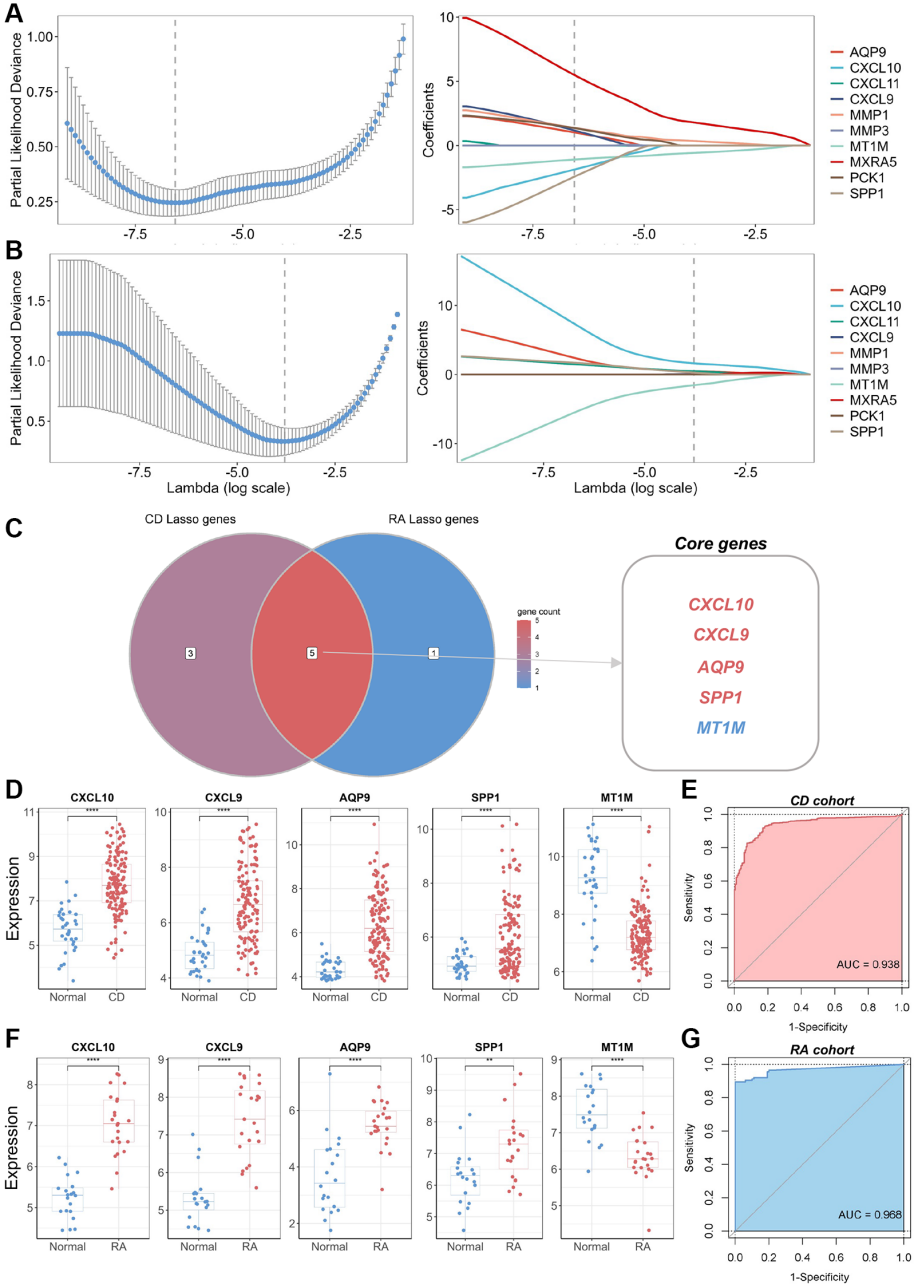
**Identification of core genes and construction of a biomarker panel for the diagnosis of CD and RA.** (**A**) Graph depicting the best LASSO model parameters and coefficients in the CD discovery cohort. (**B**) Graph depicting the best LASSO model parameters and coefficients in the RA discovery cohort. (**C**) Venn diagram displaying core genes in the CD and RA discovery cohorts. (**D**) Differential expression analysis of five core genes in the CD discovery cohort. (**E**) ROC analysis and AUC calculation for determining the diagnostic utility of a biomarker panel consisting of the five core genes in CD. (**F**) Differential expression analysis of five core genes in the RA discovery cohort. (**G**) ROC analysis and AUC calculation for determining the diagnostic utility of a biomarker panel consisting of the five core genes in RA. Abbreviations: LASSO: least absolute shrinkage and selection operator; CD: Crohn’s disease; RA: rheumatoid arthritis; ROC: receiver operating characteristic; AUC: area under the curve. ^**^P < 0.01; ^****^P < 0.0001.

To identify the core DEGs implicated in the pathological manifestations of CD and RA, we performed LASSO regression with 10-fold cross-validation using the common DEGs identified. The core genes for CD and RA were identified based on the best lambda values obtained (Figure 3A, 3B). We identified five genes (*CXCL10*, *CXCL9*, *AQP9*, *SPP1*, and *MT1M*) that were present in both CD and RA in the best LASSO model when comparing the results (Figure 3C). Therefore, we considered these five genes as core DEGs and formed a novel biomarker panel for subsequent analysis. Expression analysis of these core DEGs in the biomarker panel showed significantly higher levels of *CXCL10*, *CXCL9*, *AQP9*, and *SPP1* in lesion tissues compared to normal tissues (Figure 3D, 3F). In contrast, *MT1M* expression was significantly lower in lesion tissues. To assess the diagnostic utility of the biomarker panel for CD and RA, we conducted ROC analysis, which suggested that the biomarker panel exhibited outstanding discriminatory performance in the discovery cohort (AUC = 0.938 and 0.968, respectively) (Figure 3E, 3G). These results underscore the pivotal roles played by these biomarkers in the pathogenesis of CD and RA. However, we found that the expression levels of these core biomarkers in several other inflammatory diseases, such as UC and osteoarthritis (OA), were not consistent with those in the CD and RA cohorts (Supplementary Figure 1).

### Identification of molecular subtypes based on the biomarker panel and functional enrichment analysis

**Figure 4:**
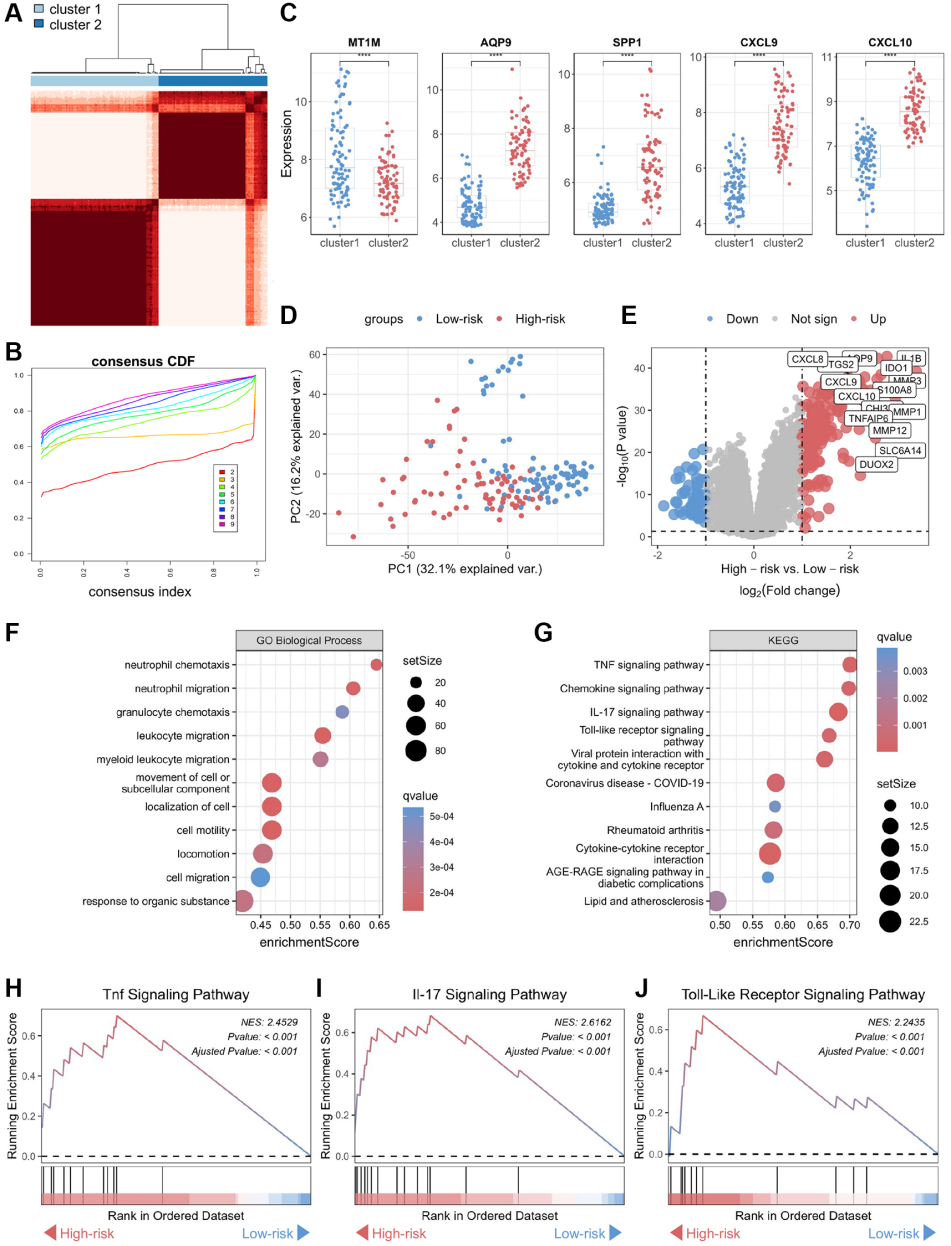
**Functional annotation of core genes in the biomarker panel based on consensus clustering in CD.** (**A**) Consensus score matrix of all samples when the number of clusters (k) is 2. (**B**) CDF curves of the consistency matrix for each k-value. (**C**) Differential analysis of the five core genes in the two subtypes (cluster 1 and cluster 2) obtained by consensus clustering. (**D**) Unsupervised PCA plots of samples in two new groups (high-risk and low-risk). (**E**) Volcano plots of DEGs in the high-risk and low-risk groups. (**F**) Gene ontology biological process and (**G**) KEGG enrichment analysis of DEGs between the high-risk and low-risk groups. GSEA of (**H**) TNF, (**I**) IL-17, and (**J**) Toll-like receptor signaling pathways. Abbreviations: CDF: cumulative distribution function; PCA: principal component analysis; KEGG: Kyoto Encyclopedia of Genes and Genomes; DEG: differentially expressed gene; GSEA: gene set enrichment analysis; TNF: tumor necrosis factor. ^****^P < 0.0001.

To further explore the involvement of the core biomarkers in disease pathogenesis, we subdivided the CD cohort into two novel CD subtypes, namely cluster 1 and cluster 2, based on a consensus clustering approach applied to the biomarker panel (Figure 4A, 4B). Subsequently, we verified the expression of these core biomarkers in both subtypes, and the results showed that the expression levels of four genes — *CXCL10*, *CXCL9*, *AQP9*, and *SPP1* — were significantly higher in cluster 2 than in cluster 1 (Figure 4C). In contrast, *MT1M* displayed a significantly lower expression level in cluster 1 as opposed to cluster 2, consistent with the expression patterns of these core biomarkers in normal and CD samples. Therefore, cluster 2 was defined as “high-risk” for CD occurrence and progression, while cluster 1 was categorized as “low-risk.” Furthermore, an unsupervised clustering PCA demonstrated a significant difference between the high-risk and low-risk groups (Figure 4D). A volcano plot showcased differences in gene expression between these two groups, highlighting genes with significantly elevated expression levels in the high-risk group (Figure 4E). Functional annotation of the DEGs showed that the key biological processes linked to the biomarker panel identified in this study encompassed neutrophil chemotaxis, neutrophil migration, granulocyte chemotaxis, and leukocyte migration (Figure 4F). Additionally, KEGG analysis showed that tumor necrosis factor (TNF), chemokine, interleukin-17 (IL-17), and toll-like receptor (TLR) signaling pathways are most closely associated with the biomarker panel (Figure 4G). Furthermore, GSEA demonstrated significant upregulation of TNF, IL-17, and TLR signaling pathways in the high-risk group compared to the low-risk group (Figure 4H–4J).

We performed the same analysis in the RA cohort and discovered that the biomarker panel identified in this study was associated with RA progression (Supplementary Figure 2).

### Immune infiltration analysis of molecular subtypes based on the biomarker panel

**Figure 5:**
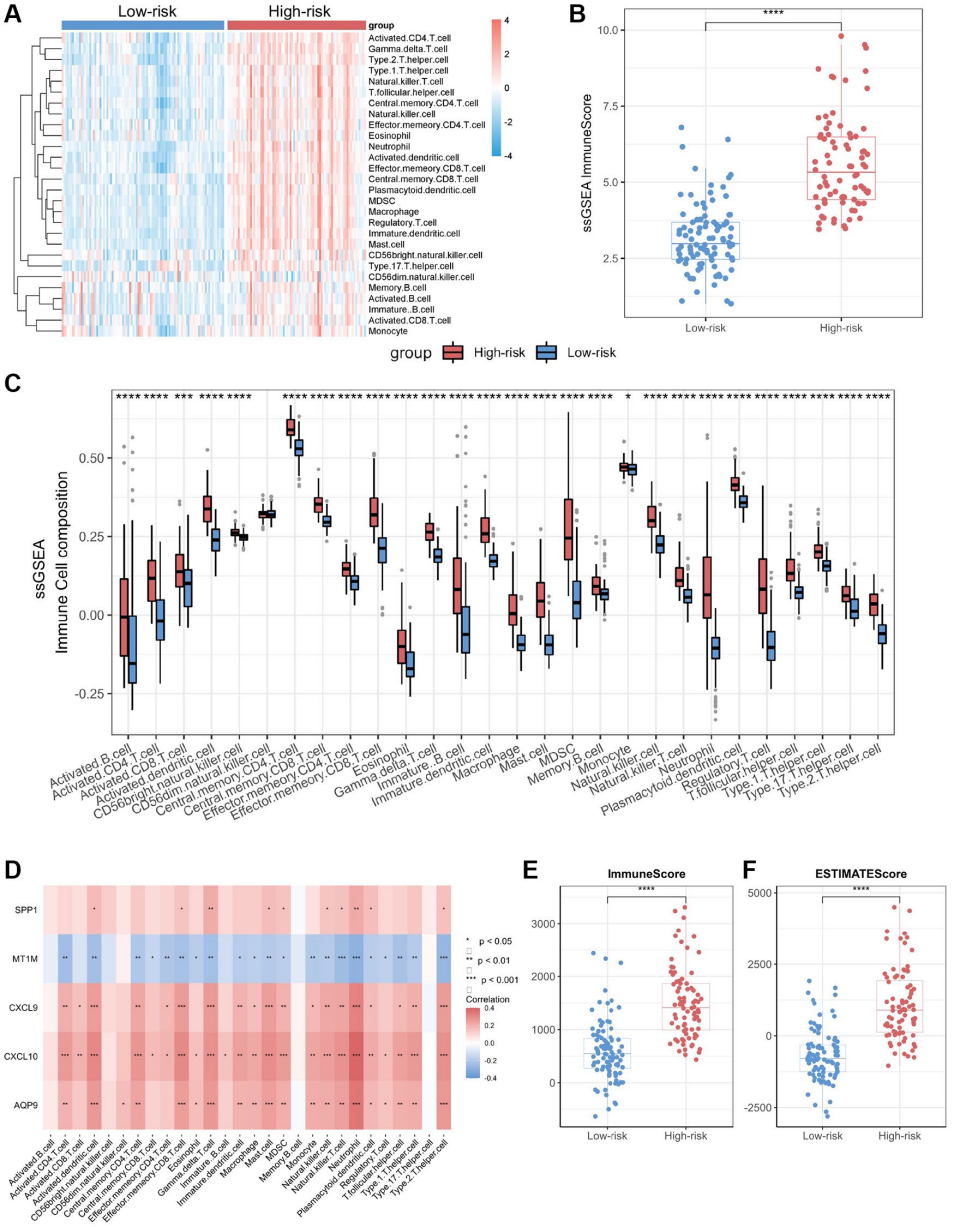
**Immuno-infiltration analysis of core genes in CD.** (**A**) Heatmap of immune cell type scores based on ssGSEA. (**B**) Differential analysis of the sum of immune scores based on ssGSEA. (**C**) Differential analysis of immune cell type scores based on ssGSEA. (**D**) Correlation heatmap of core genes and immune cell types. (**E**) Differential analysis of immune scores based on ESTIMATE. (**F**) Differential analysis of ESTIMATE scores. Abbreviation: ssGSEA: single-sample gene set enrichment analysis. ^*^P < 0.05; ^**^P < 0.01; ^***^P < 0.001; ^****^P < 0.0001.

We performed immune infiltration analyses on CD subtypes using the ssGSEA algorithm. The results showed significant differences in immune cell infiltration levels and immune scores between the high-risk and low-risk groups (Figure 5A, 5B). The bowel tissues from the high-risk group exhibited higher levels of immune cell infiltration, including macrophages, neutrophils, T helper type 1 (Th1) cells and T helper type 17 (Th17) cells, than those in the bowel tissues from the low-risk group (Figure 5C). Employing Spearman correlation analysis, we confirmed that the expression levels of *CXCL10*, *CXCL9*, *AQP9*, and *SPP1* were significantly and positively correlated with the numbers of most immune cell types in the immune infiltrates; however, the most significant correlation was observed with neutrophils (Figure 5D). In contrast, *MT1M* expression levels exhibited a negative correlation with immune cell infiltration levels. In addition, we assessed immune infiltration levels in the high-risk and low-risk groups using the ESTIMATE and CIBERSORT algorithms. The results were consistent with those obtained through the ssGSEA algorithm, the high-risk group displayed higher levels of immune infiltration compared to those from the low-risk group (Figure 5E, 5F; Supplementary Figure 3).

In addition, we validated the relationship between the biomarker panel and immune infiltration levels in the RA cohort and reaffirmed the elevated immune infiltration levels in the high-risk group (Supplementary Figure 4).

### Evaluation of the diagnostic significance of the biomarker panel in validation cohort

**Figure 6:**
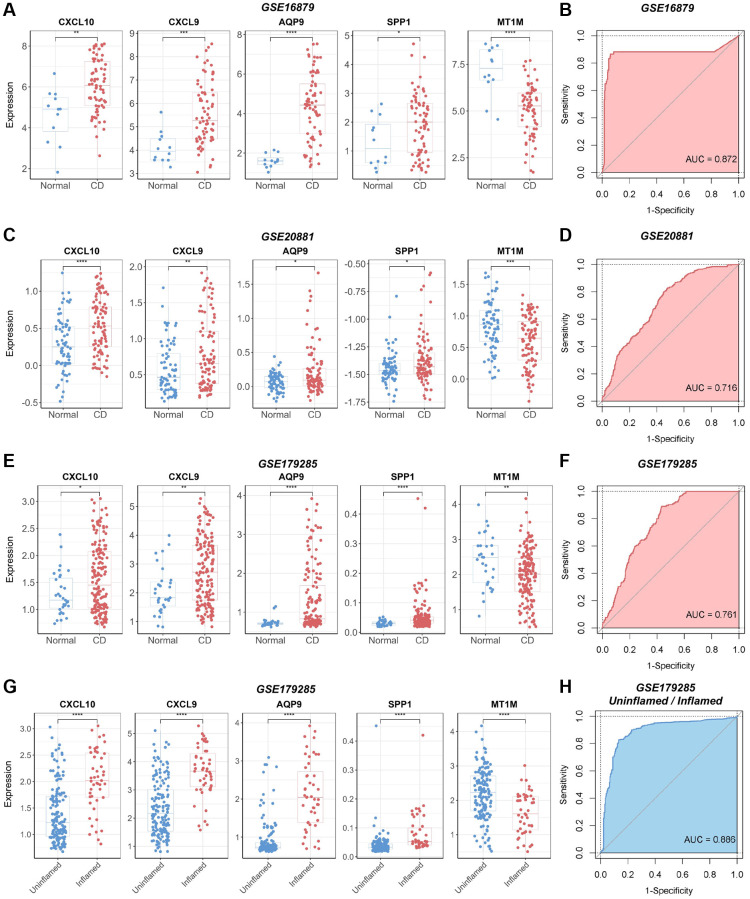
**Verification of the diagnostic utility of the biomarker panel in CD validation cohorts.** (**A**) Expression analysis and (**B**) ROC curve analysis of core genes in the GSE16879 dataset. (**C**) Expression analysis and (**D**) ROC curve analysis of core genes in the GSE20881 dataset. (**E**) Expression analysis and (**F**) ROC curve analysis of core genes in the GSE179285 dataset. (**G**) Core gene expression analysis and (**H**) ROC curve analysis of samples with and without inflammation in the GSE179285 dataset. Abbreviations: ROC: receiver operating characteristic; AUC: area under the curve. ^*^P < 0.05; ^**^P < 0.01; ^***^P < 0.001; ^****^P < 0.0001.

We validated the identified biomarkers by assessing the expression levels of the genes in several independent GEO datasets. The results indicated elevated expression levels of *CXCL10*, *CXCL9*, *AQP9*, and *SPP1* in CD samples across the CD datasets GSE16879, GSE20881, and GSE179285, aligning with our initial findings in the discovery CD cohort (Figure 6A, 6C, 6E). In contrast, *MT1M* exhibited reduced expression levels in CD samples compared to normal samples. All selected biomarkers exhibited robust discriminatory performance between CD samples and normal samples (AUC = 0.872, AUC = 0.716, and AUC = 0.761 for GSE16879, GSE20881, and GSE179285, respectively), as determined through 10-fold cross-validated ROC analysis (Figure 6B, 6D, 6F). In the GSE179285 dataset, *CXCL10*, *CXCL9*, *AQP9*, and *SPP1* displayed increased expression in samples with inflammation, while *MT1M* exhibited higher expression levels in samples without inflammation (Figure 6G). The selected biomarkers also demonstrated strong discriminative performance between samples with inflammation and those without inflammation (AUC = 0.886) (Figure 6H).

In addition, we validated the identified biomarkers for RA diagnosis using the GSE77298 dataset. The results demonstrated the efficacy of the selected biomarkers in distinguishing RA samples from normal samples in this validation cohort (AUC = 0.794) (Supplementary Figure 5).

### Construction of biomarker interaction networks and identification of therapeutic compounds

**Figure 7:**
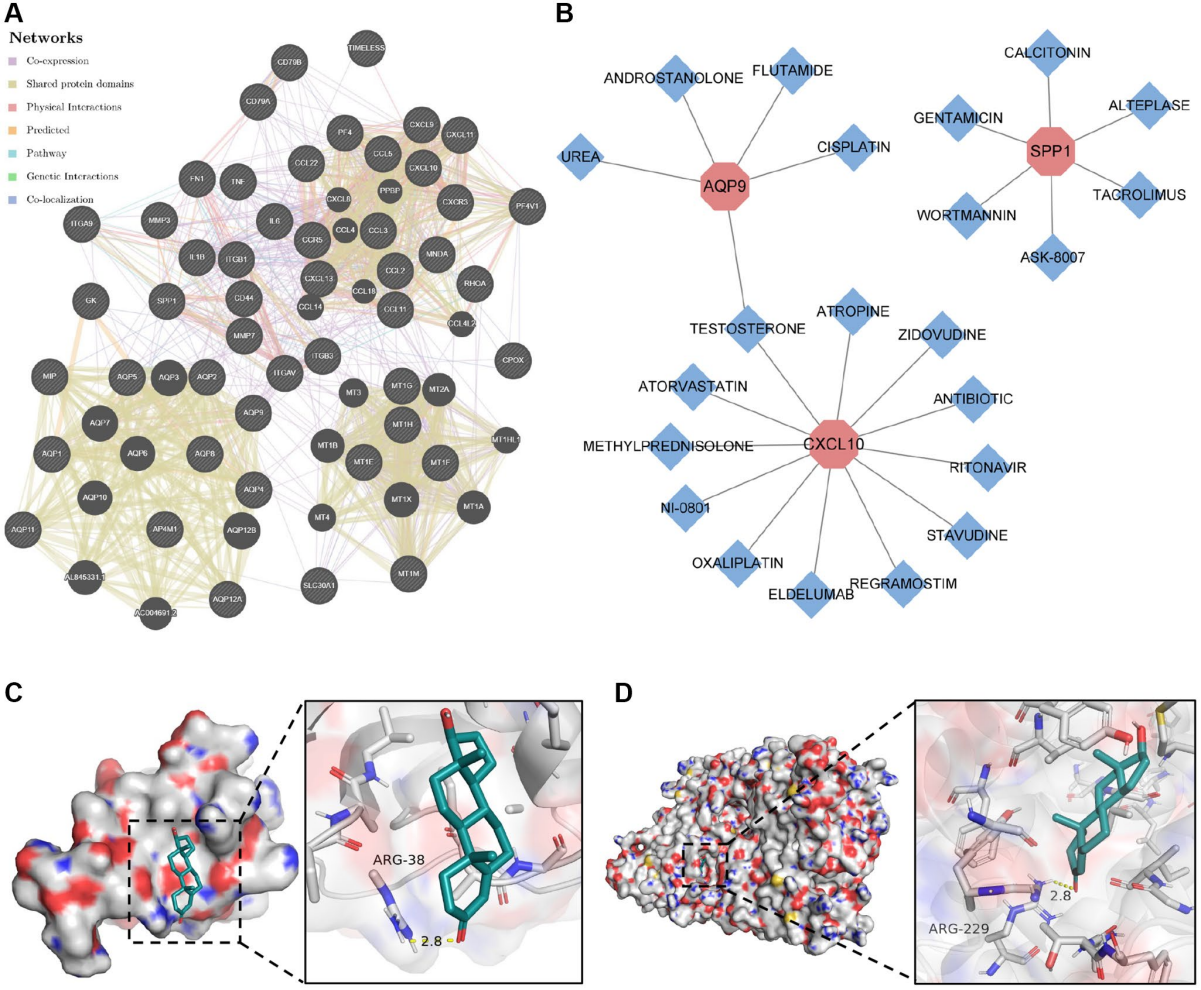
**Construction of protein–protein interaction network and drug–gene action network.** (**A**) Protein–protein interaction network of core genes. (**B**) Drug–gene interaction network of core genes. (**C**) Molecular docking of testosterone binding to CXCL10. (**D**) Molecular docking of testosterone binding to AQP9.

To elucidate the molecular mechanisms underlying the influence of the selected biomarkers on disease pathology, we initially constructed an miRNA–mRNA co-expression network for these biomarkers. The results showed that 55 miRNAs possessed the potential to target these core biomarkers. Among these, miR-181c-5p emerged as a candidate capable of targeting both *CXCL9* and *SPP1* (Supplementary Figure 6). In addition, we established a protein–protein interaction network by leveraging data from the STRING and GeneMANIA databases (Figure 7A). Subsequently, we employed the DGI database to identify compounds with the potential to inhibit the functions of these biomarkers, leading to the identification of 22 compounds targeting CXCL10, AQP9, and SPP1 proteins (Figure 7B). Among these compounds, testosterone emerged as a potential co-acting compound with the ability to target AQP9 and CXCL10. The binding of testosterone to CXCL10 and AQP9 was subsequently confirmed through molecular docking simulations. The results showed that testosterone formed hydrogen bonds with Arg-38 in CXCL10 at a distance of 2.8 Å (Figure 7C) and with Arg-229 in AQP9 at a similar distance of 2.8 Å (Figure 7D). These results suggest that testosterone can be used to target CXCL10 and AQP9.

### Validation of genes in the biomarker panel by q-PCR

**Figure 8:**
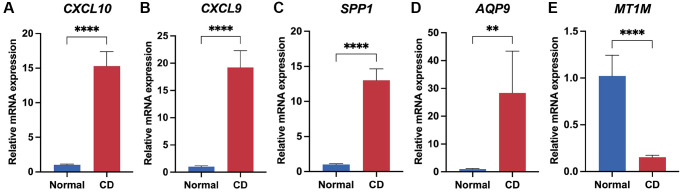
**Validation of biomarker panel expression in CD lesions and normal tissues by q-PCR.** Differential comparison of mRNA expression levels of CXCL10 (**A**), CXCL9 (**B**), SPP1 (**C**), AQP9 (**D**), and MT1M (**E**) in CD and normal tissues. ^**^P < 0.01; ^****^P < 0.0001.

To validate the expression of the biomarker panel, we performed q-PCR on both CD lesions and normal intestinal tissues. The human intestinal tissues utilized in this research were sourced from patients undergoing intestinal resection at the Department of Gastrointestinal Surgery, Shandong Provincial Third Hospital, with all patients consenting through signed informed consent forms. The Crohn's Disease (CD) lesion tissues were obtained from CD patients who underwent local intestinal resection, while the normal intestinal tissues were obtained from constipated patients who underwent total colectomy. The results demonstrated significant upregulation of four genes, specifically *CXCL10, CXCL9, AQP9*, and *SPP1*, in CD-afflicted tissues (*P* < 0.05) (Figure 8A–8D). Conversely, *MT1M* exhibited heightened expression levels in normal tissues (*P* < 0.05) (Figure 8E). Furthermore, the expression of the five genes in the biomarker panel was verified using serum samples from patients with and without RA. The results indicated elevated levels of *CXCL10, CXCL9, AQP9*, and *SPP1* in the serum of patients with RA (*P* < 0.05), while *M1TM* demonstrated lower expression in the serum of patients with RA (*P* < 0.05) (Supplementary Figure 7). These results were consistent with the transcriptomic data, affirming the reliability of the biomarker panel.

## DISCUSSION

In this study, we identified shared core genes between CD and RA. The primary findings are as follows: (1) A biomarker panel comprising common core genes, specifically *CXCL10*, *CXCL9*, *AQP9*, *SPP1*, and *MT1M*, effectively distinguished CD/RA samples from normal samples in both discovery and validation cohorts. (2) Utilizing the biomarker panel, we observed significantly higher levels of immune cell infiltration in the high-risk groups compared to the low-risk groups. (3) Testosterone emerged as a potential therapeutic agent for CD and RA, as it exhibited the capacity to target some of the common core biomarkers in the biomarker panel.

Recently, both CD and RA have gained recognition as immune-mediated inflammatory conditions that frequently induce systemic inflammation [18, 19]. CD and RA are frequently treated with the same medications, implying common underlying pathological processes [4, 20]. To investigate the similarities between CD and RA, we conducted bioinformatics analysis and found that several genes are differentially expressed (compared to normal tissues) in both CD and RA. Among the several DEGs identified in this study, five genes, namely *CXCL10*, *CXCL9*, *AQP9*, *SPP1*, and *MT1M*, were identified as core genes. Furthermore, we found that the biomarker profiles of these core genes exhibited the same expression patterns in the validation cohorts of both CD and RA. Moreover, these patterns were not observed in other types of inflammatory disease cohorts, such as UC and OA. These findings confirmed that the selected biomarkers exhibit specific expression patterns in CD and RA.

Several studies have suggested that CXCL10 and CXCL9 can serve as markers of disease activity in both CD and RA and play roles in the pathogenesis of these diseases [21, 22]. Several drugs targeting the CXCL10 protein have undergone clinical trials as potential treatment for autoimmune and inflammatory diseases [23]. In the pathophysiology of CD, CXCL10 functions as a ligand for the CXCR3 receptor, triggering the recruitment of T lymphocytes and contributing to the persistence of mucosal inflammation [24]. In RA, CXCL10 is primarily expressed by macrophage-like cells and fibroblast-like synoviocytes (FLS) infiltrating the synovium. The interaction between CXCL10 and the nuclear factor kappa-B ligand (RANKL), as well as other cytokines (e.g., TNF-α), may initiate and/or aggravate inflammation and bone erosion in RA [25]. Similar to CXCL10, CXCL9 serves as a ligand for CXCR3, facilitating the aggregation of T cells within the inflamed intestine regions in CD and Th1 cells in the synovium in RA [26, 27]. These findings suggest that both CXCL9 and CXCL10 may foster the progression of inflammation by promoting immune cell aggregation in CD and RA. These findings are consistent with the results of our immune infiltration analyses conducted in this study, further reinforcing the potential usefulness of the biomarker panel.

AQP9 is involved in the pathogenesis of various inflammatory diseases and holds promise as a potential biomarker for clinical diagnosis [28, 29]. Previous studies have shown an upregulation in the mRNA and protein expression of AQP9 in human neutrophils and primary blood-derived macrophages following lipopolysaccharide stimulation [30]. AQP9 has been suggested to play a role in leukocyte motility by facilitating cell extension and stabilizing the lamellar substrate [31]. Additionally, it is involved in the regulation of cellular volume changes, a crucial factor enabling leukocytes to effectively migrate towards chemoattractants [31]. A previous study also considered AQP9 as a universal inflammatory marker for the diagnosis of CD and RA, which aligns with our findings [32].

The *SPP1* gene encodes human osteopontin (OPN), an arginine-glycine-aspartate domain-containing phosphoprotein predominantly expressed in epithelial cells, activated T cells, macrophages, and osteoblasts [33]. Previous studies have demonstrated the production of OPN by plasma cells in the mucosa of CD lesions, where it acts as a potent IL-12 inducer in CD intestinal mucosal macrophages, playing a crucial role in establishing the Th1 cytokine milieu necessary for chronic inflammation in CD [34]. In the context of RA pathology, FLS produce OPN at sites of cartilage invasion and in the synovial lining [35]. This leads to the attachment of FLS to cartilage and the production of MMP1 in chondrocytes, which, in turn, promotes the degradation of the extracellular matrix. In addition, OPN may contribute to the induction and onset of arthritis by polarizing the Th1 cytokine response and fostering bone resorption by osteoclasts [36]. The results of these studies indicate that SPP1 is predominantly expressed in immune cells within diseased tissues in both CD and RA. This suggests that SPP1 may have significant diagnostic value and is closely associated with immune infiltration, a finding consistent with our own findings.

*MT1M* encodes a protein belonging to the cysteine-rich metallothionein family, known for its ability to bind heavy metals via cysteine thiol groups [37]. Reduced levels of MT1M increase oxidative stress by altering the regulation of superoxide radicals and indirectly by affecting zinc metabolism. Low MT1M levels lead to a redox imbalance and an inability to maintain an adequate reducing environment, which in turn leads to oxidative stress [38]. Previous studies have suggested an association between MT1M and cuproptosis, noting lower expression levels in CD tissues than in normal tissues, as well as a negative correlation with immune cell infiltration [39]. Consistent with these previous findings, MT1M expression was also reduced in CD lesion tissues in this study. In addition, we also found a decrease in MT1M expression in RA lesion tissues; however, these findings warrant further validation.

Aberrant TNF signaling has been thought to play a significant role in the pathogenesis of inflammatory diseases such as CD and RA [40, 41]. Previous studies have demonstrated that the IL-17 signaling pathway is closely linked to the pathogenesis of CD [42]. Similarly, excessive activation of TLR signaling has been suggested as a risk factor for CD [43]. In this study, we found that biomarkers common to CD and RA were associated with the activation of the TNF, IL-17, and TLR signaling pathways in the CD cohort. Additionally, in the RA cohort, we found that these biomarkers activated RA signaling and immune response signaling pathways. These findings lend theoretical support to the notion that extraintestinal arthropathy in CD may be linked to RA and corroborate the pathological significance of the biomarkers identified in this study in both RA and CD.

In patients with CD, there is a generalized increase in immune cell infiltration and activation in the intestinal mucosa, which is thought to exacerbate local inflammation [44, 45]. In this study, we discovered that the high-risk group of patients with CD exhibited higher levels of immune infiltration and higher expression levels of the identified biomarkers compared to the low-risk group. This suggests that the biomarkers identified in this study may play a role in modulating immune activity in the CD microenvironment. Similarly, the pathological manifestations of RA are primarily caused by infiltrating immune cells [46, 47]. We found that the high-risk group of patients with RA also displayed higher expression levels of the biomarkers identified in this study, along with heightened immune infiltration scores, compared to the low-risk group. The high-risk groups exhibited active immune infiltrates, aligning with the immune activity in the pathological processes of both CD and RA. These findings suggest that the immune mechanisms underlying CD and RA may share similarities and further imply that the identified biomarkers exert an influence on the altered immune activity in the pathological processes.

Previous studies have reported the significant role of miRNAs in the development and progression of both CD and RA [19, 48]. Therefore, we constructed miRNA–mRNA co-expression networks to investigate the molecular regulatory mechanisms of the identified biomarkers at the transcriptional level. We found that 55 miRNAs exhibited co-expression with the core biomarkers, shedding light on potential regulatory mechanisms for a more in-depth investigation of these diseases. In addition, the protein–protein interaction network we constructed revealed that the regulatory mechanisms underlying the common biomarkers of CD and RA are linked to TNF, IL-1β, and IL-6 signaling pathways. These pro-inflammatory factors play a crucial pathogenic role in the development of CD [49, 50]. Our findings potentially provide novel theoretical support for the existence of a shared molecular mechanism connecting the pathogenesis of CD and RA, particularly in the context of extra-intestinal arthropathy in CD.

Recently, the same therapeutic agents have often been employed to treat both CD and RA [4, 51], confirming the existence of similar pathologies in CD and RA. This also suggests that drug discovery based on common pathological alterations shared between these two diseases may yield more rational choices for clinical treatment. In this study, we found that testosterone can target both CXCL10 and AQP9 proteins, which were identified as common biomarkers for CD and RA. In addition, the binding of testosterone to CXCL10 and AQP9 was confirmed through molecular docking. According to previous reports, serum testosterone levels are reduced in patients with inflammatory bowel disease, and testosterone treatment can alleviate inflammation and reduce disease burden by inhibiting the expression of pro-inflammatory cytokines [52, 53]. Thus, our findings provide theoretical support for using testosterone as a potential treatment option for CD and RA, especially in cases of extra-intestinal arthropathy associated with CD.

In this study, we highlighted the potential role of core biomarkers common to CD and RA. Nonetheless, our study has a few limitations, which we briefly delineate as follows: (1) Despite the relatively large sample size of the discovery cohort and the validation of the expression and diagnostic utility of the core genes across other datasets, it remains imperative to conduct external validation. (2) The biological functions of the core genes need to be more comprehensively validated using *in vivo* and *in vitro* models. Our forthcoming research endeavors will be directed towards addressing this aspect.

To the best of our knowledge, our study investigated the coexistence of pathogenic and protective molecules in both CD and RA for the first time. Additionally, we utilized the LASSO algorithm to identify core genes and subsequently constructed a novel biomarker panel. More notably, these molecules may exert their effects on the disease process by modulating the type and quantity of immune cells within the lesion tissues of both CD and RA, either facilitating an increase or decrease. Indeed, in multiple validation cohorts, we observed that the novel biomarker panel comprising these molecules exhibited excellent diagnostic performance for both CD and RA. Furthermore, this was confirmed by conducting validation experiments using CD tissue samples and RA serum samples. In conclusion, our study offers partial theoretical support for the existence of similar molecular mechanisms in both CD and RA, thereby providing novel insights into the diagnosis and treatment of these diseases.

## Supplementary Materials

Supplementary Figures
