# Supplementary material for: Development and validation of a novel biomarker panel for Crohn’s disease and rheumatoid arthritis diagnosis and treatment
Source: Aging (Albany NY). 2024 Mar 10;16(6):5224–48. doi: 10.18632/aging.205644 (PMC11006481; doi:10.18632/aging.205644)
Supplement: Supplementary Figures [file aging-16-205644-s001.pdf]

SUPPLEMENTARY FIGURES

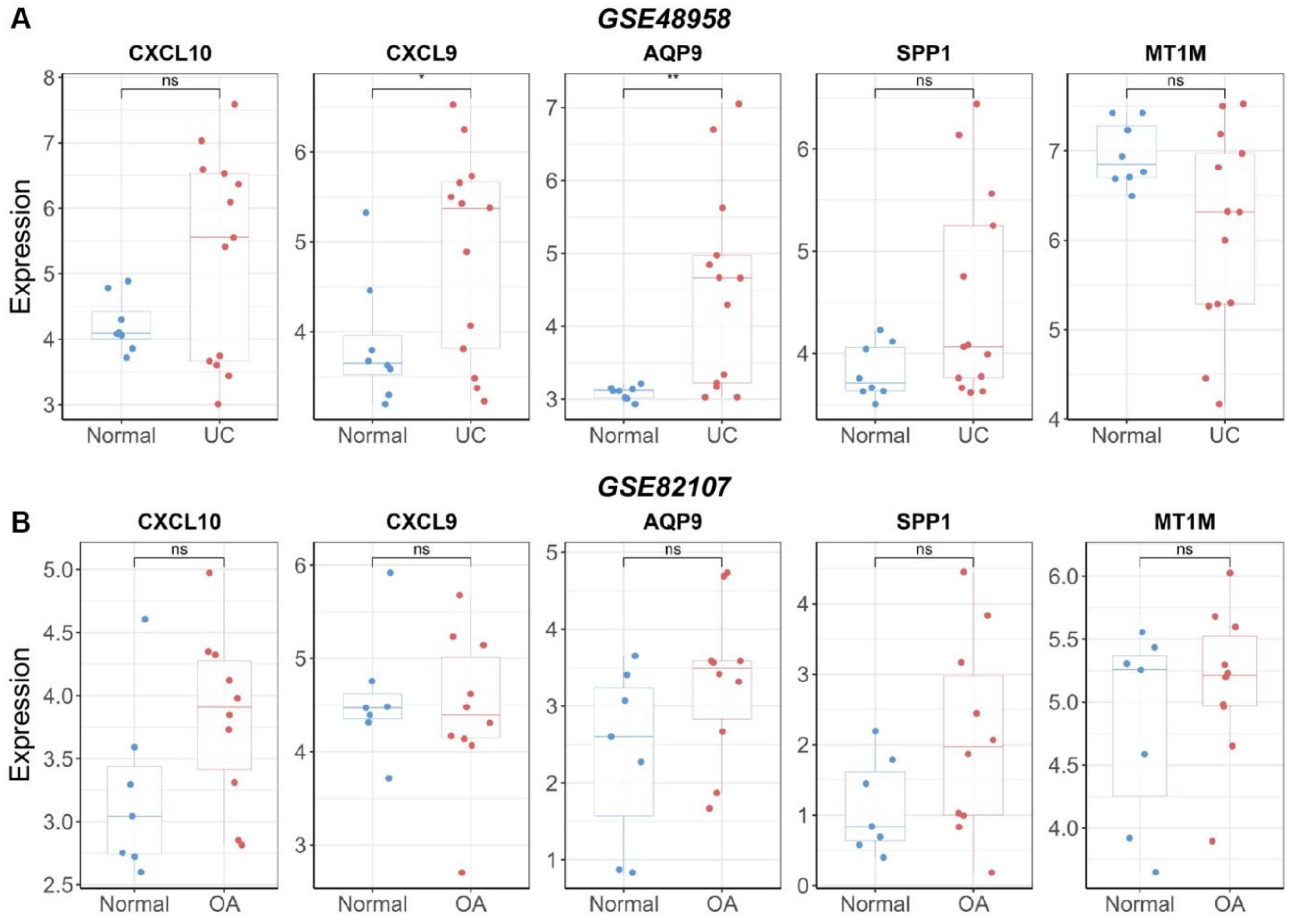

Supplementary Figure 1. Validate the expression of the core genes in the UC and OA cohorts. (**A**) Differential expression analysis of 5 core genes in the UC cohort. (**B**) Differential expression analysis of 5 core genes in the OA cohort. Abbreviations: UC: ulcerative colitis; OA: osteoarthritis; ns: no significance. $^{*}P < 0.05$ $^{**}P < 0.01$.

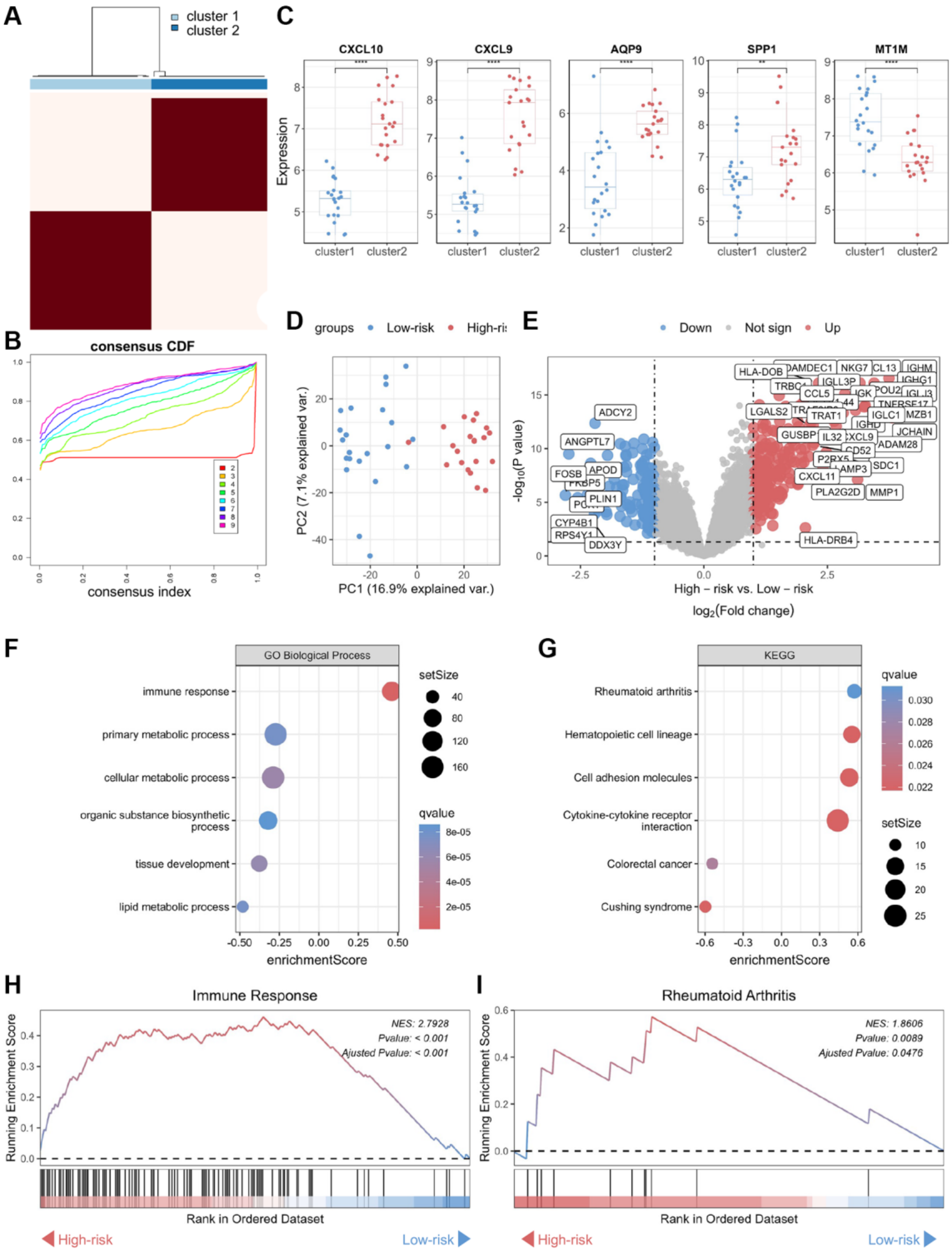

Supplementary Figure 2. Functional annotation of core genes in the biomarker panel based on consensus clustering in RA. (**A**) Consensus score matrix of all samples when the number of clusters k = 2. (**B**) CDF curves of the consistency matrix for each k-value. (**C**) Differential analysis of the five core genes in the two subtypes (cluster 1 and cluster 2) obtained by consensus clustering. (**D**) Unsupervised PCA plots of samples in two new groups (high-risk and low-risk). (**E**) Volcanoes of differentially expressed genes in high-risk and low-risk group. (**F**) Gene ontology biological process and (**G**) KEGG enrichment analysis of DEGs between high-risk and low-risk. GSEA analysis of (**H**) Immune Response, and (**I**) Rheumatoid Arthritis signaling pathways. Abbreviations: CDF: cumulative distribution function; PCA: Principal Component Analysis; KEGG: Kyoto Encyclopedia of Genes and Genomes; DEG: Differentially Expressed Gene; GSEA: Gene Set Enrichment Analysis; TNF: tumor necrosis factor. $^{**}P < 0.01$, $^{****}P < 0.0001$.

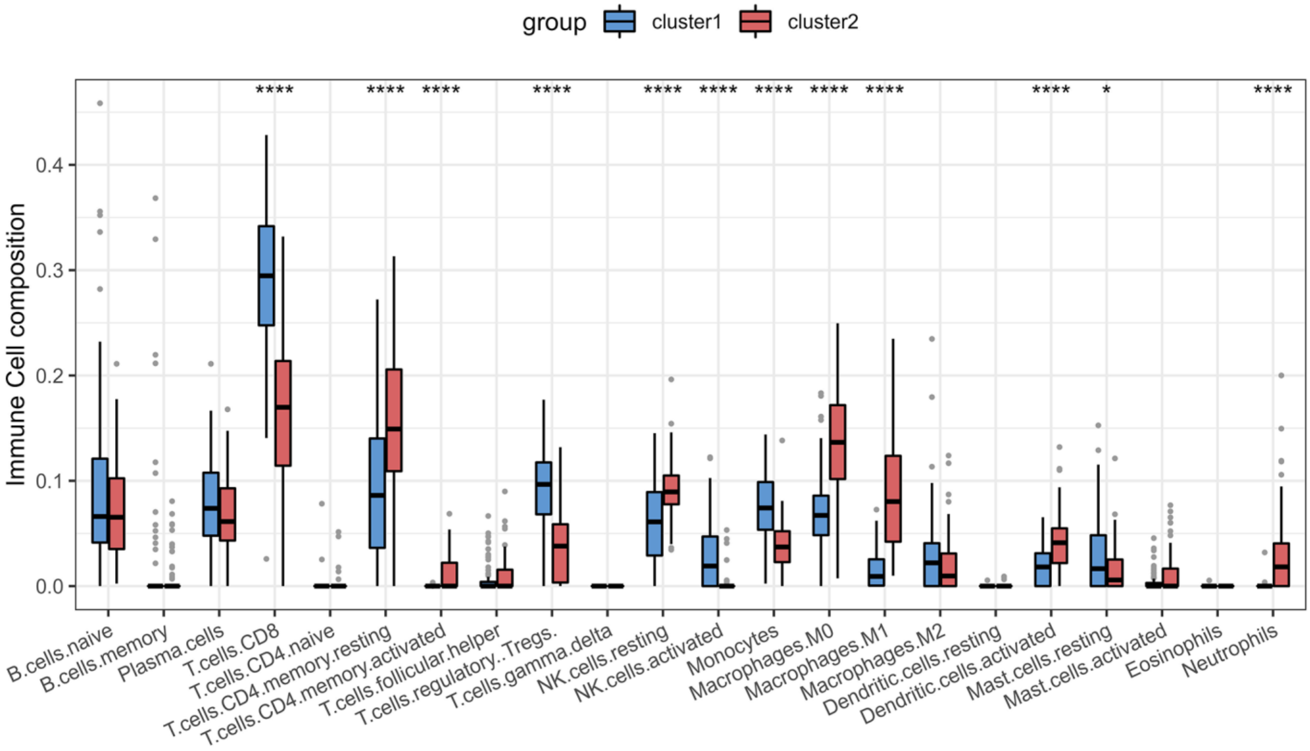

Supplementary Figure 3. Differential analysis of immune cell type scores based on CIBERSORT. $^{*}P < 0.05$; $^{****}P < 0.0001$.

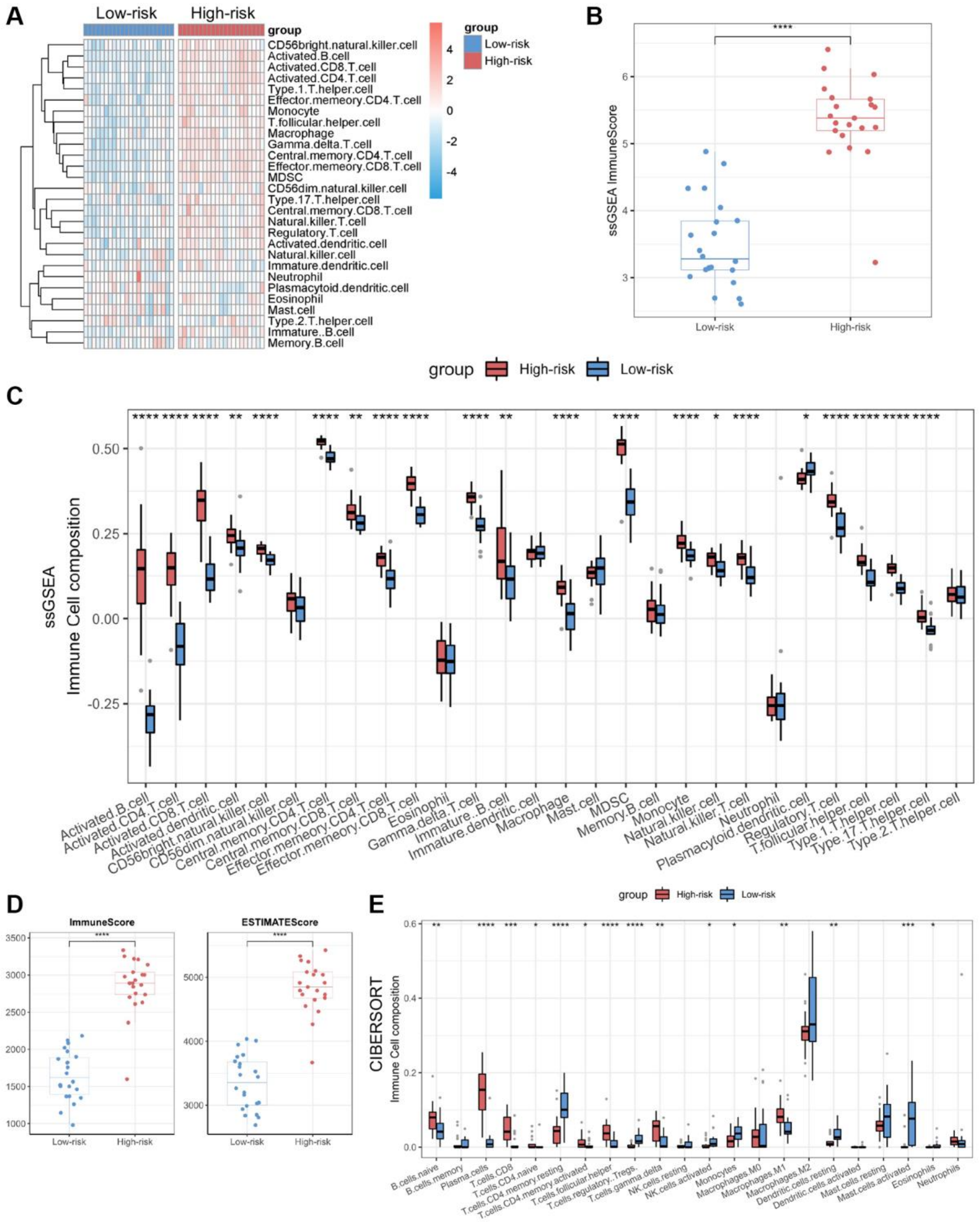

Supplementary Figure 4. Immuno-infiltration analysis of core genes in RA. (**A**) Heat map of immune cell type scores based on ssGSEA. (**B**) Differential analysis of the sum of immune scores based on ssGSEA. (**C**) Differential analysis of immune cell type scores based on ssGSEA. (**D**) Differential analysis of ESTIMATE-based immune scores and ESTIMATE score. (**E**) Differential analysis of immune cell type scores based on CIBERSORT. Abbreviation: ssGSEA: single sample Gene Set Enrichment Analysis. $^{*}P < 0.05$; $^{**}P < 0.01$; $^{***}P < 0.001$; $^{****}P < 0.0001$.

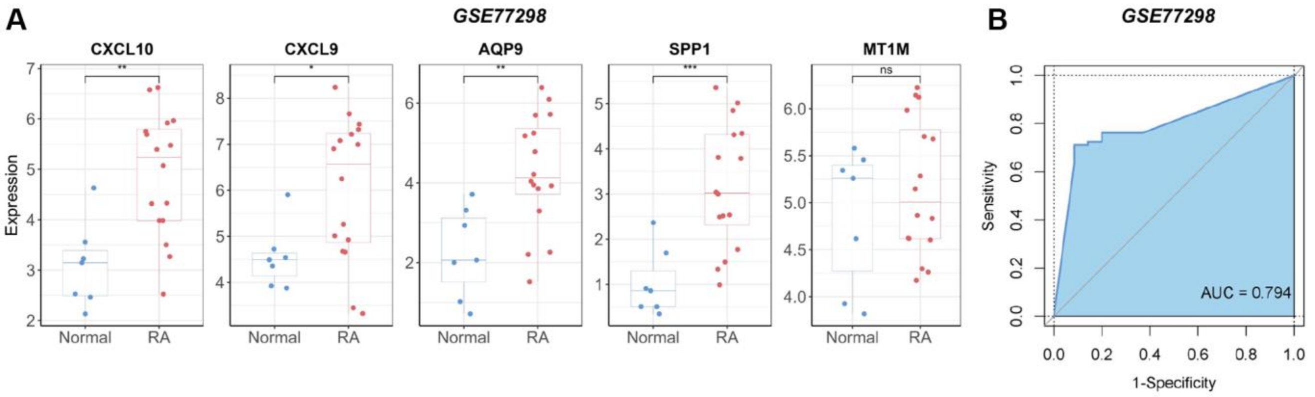

Supplementary Figure 5. Verification of the diagnostic value of the biomarker panel in RA validation cohorts. (**A**) Expression analysis and (**B**) ROC curve analysis of core genes in GSE77298. Abbreviations: ROC: Receiver Operating Characteristic; AUC: Area Under Curve; ns: no significance. $^{*}P < 0.05$; $^{**}P < 0.01$; $^{***}P < 0.001$.

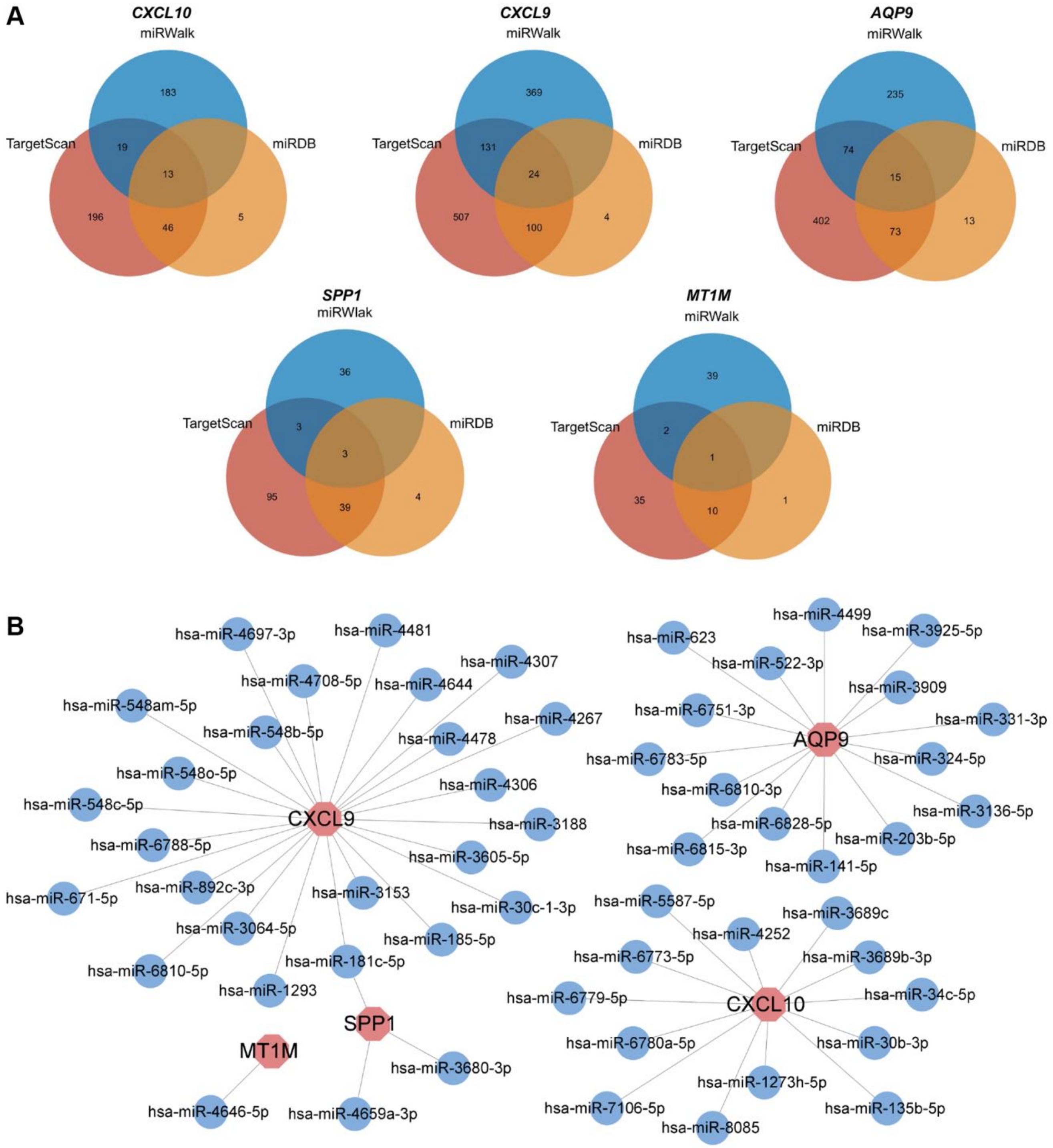

Supplementary Figure 6. Construction of miRNA-mRNA co-expression network. (**A**) Acquisition of core gene co-expression miRNA Venn diagrams based on TargetScan, miRWalk, and miRDB. (**B**) Visualization of co-expression networks of miRNAs and core genes.

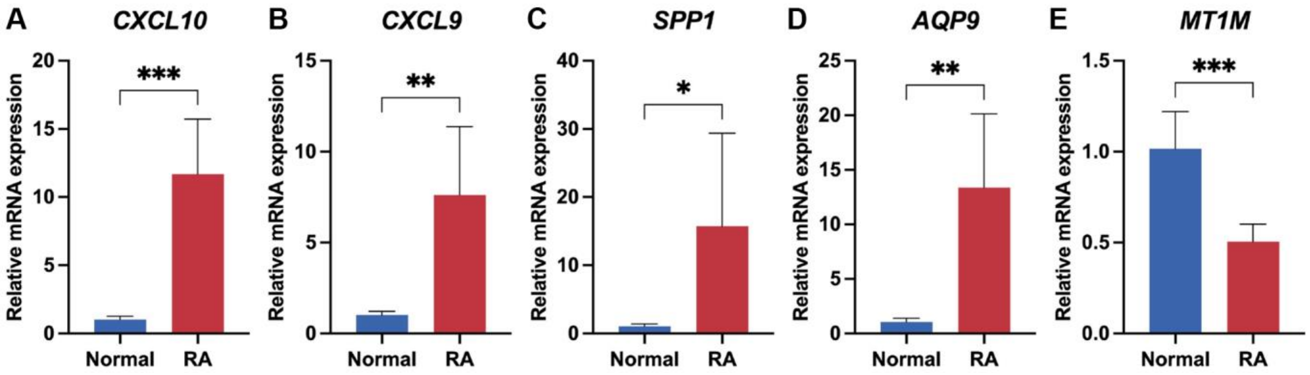

Supplementary Figure 7. Validation of biomarker panel expression by q-PCR in serum samples from patients with RA and healthy controls. Differential comparison of mRNA expression levels of CXCL10 (**A**), CXCL9 (**B**), SPP1 (**C**), AQP9 (**D**), MT1M (**E**) in serum samples from patients with RA and healthy controls. $^{*}P < 0.05$; $^{**}P < 0.01$; $^{***}P < 0.001$.
